# Long-Term Care Planning and Sustainability of the Care System in the Region

**DOI:** 10.3390/healthcare14121633

**Published:** 2026-06-09

**Authors:** Carmen Rajer, David Bogataj, Marija Bogataj, Samo Drobne

**Affiliations:** 1 Department of Social Gerontology, Alma Mater Europaea University, Slovenska 17, 2000 Maribor, Slovenia; carmen.rajer@almamater.si (C.R.); marija.bogataj@guest.arnes.si (M.B.); 2 Institute for Research of Systems Exposed to Risk, Kidričeva 1, 8210 Trebnje, Slovenia; 3 Faculty of Civil and Geodetic Engineering, University of Ljubljana, Jamova 2, 1000 Ljubljana, Slovenia

**Keywords:** long-term care, long-term home care, community-based care, ageing population, spatial accessibility, travel costs, rural areas, care preferences, integrated care, Slovenia

## Abstract

Background/Objectives: This study examines the relationship between user preferences, spatial accessibility, and the financial sustainability of long-term care (LTC) systems, with a focus on Slovenia and the Posavje region. The analysis compares different care models, including long-term home care (LTHC), institutional care, and community-based housing solutions such as sheltered housing and “silver villages”. Methods: A mixed-methods approach was applied, combining qualitative interviews, survey data, spatial analysis, mobility-related operational assessment, and cost estimation. The survey included 1005 individuals, of whom 475 provided valid responses. Statistical analysis was conducted using chi-square tests and the Agresti–Caffo method to examine differences in care preferences and selected proportions across respondent groups. Results: Statistically significant differences in LTC preferences across age groups were identified. Most respondents preferred care options located close to their homes, with the majority unwilling to relocate more than 10 km and a substantial share preferring distances below 5 km. The findings further indicate that travel-related costs for care providers in rural areas are considerable and, in the Municipality of Krško, comparable to the estimated monthly housing costs in specialized community-based units. Cost comparisons suggest that reductions in travel-related operational costs could offset a substantial share of the estimated housing-related costs. Conclusions: The results indicate that sustainable LTC systems require not only adequate service capacity and funding but also spatially coordinated care models aligned with user preferences and long-term sustainability. The findings contribute to strategic LTC planning and support the development of integrated, community-based care systems in geographically dispersed regions.

## 1. Introduction

### 1.1. Growing Share of Older Adults and Their Needs

Across Europe and other developed regions, long-term care (LTC) systems face increasing pressure from population ageing, rising demand, and growing financial constraints [1,2]. Policy responses increasingly promote integrated, long-term home care (LTHC) and community-based care models that support ageing in place [2]. While these approaches are associated with improved quality of life and reduced institutionalization, their cost-effectiveness remains context-dependent and not yet conclusively established [3,4].

Integrated care has become a central concept in LTC, combining health and social services around individual needs [5]. Core elements include multidisciplinary teams, case management, and continuity of care across providers [6]. More recently, integrated care pathways have been proposed as a structured approach to improving coordination across services and ensuring continuity of care [7]. Evidence suggests that such models can improve outcomes and access to services, although their effectiveness depends on implementation context and governance structures [8,9].

**Figure 1:**
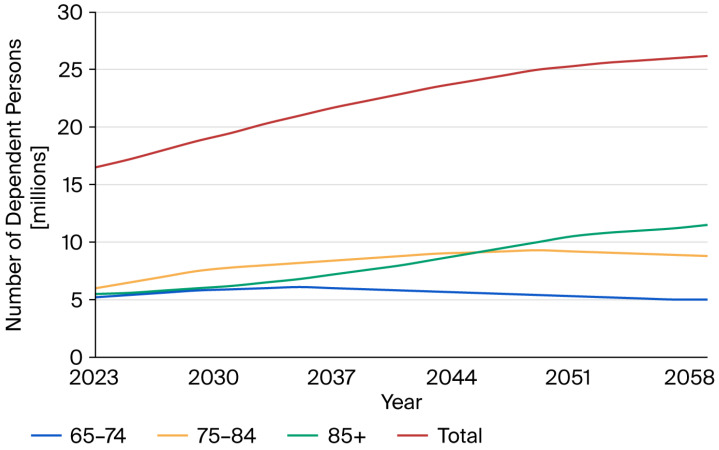
Projection of the number of dependent older adults in the EU, 2023–2060 (source: [11] and authors’ elaboration).

**Figure 2:**
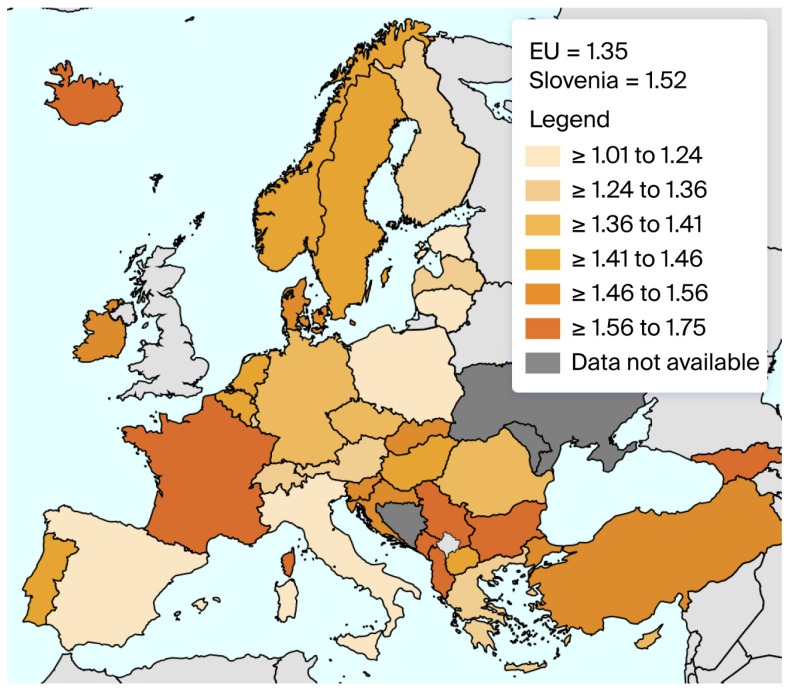
Total fertility rates in EU member states in 2024 (source: [12]).

The increasing share of older adults reflects advances in healthcare and living standards, as well as declining fertility and broader socioeconomic changes. A growing proportion of older adults require assistance with daily activities and LTC support (Figure 1). In Slovenia, population ageing is progressing faster than the EU average, primarily due to increasing longevity and demographic dynamics. Although the total fertility rate in Slovenia (1.52) exceeds the EU average (1.34 in 2024; Figure 2), it remains below the replacement level, contributing to population decline and ageing [2,10].

Figure 1 and Figure 2 provide the broader demographic context within which long-term care systems are expected to operate in the coming decades. The projected increase in the older dependent population, together with persistently low fertility rates, represents an important structural factor affecting the future accessibility, organization, and financial sustainability of LTC provision across Europe. Although Slovenia is not among the European countries facing the most extreme demographic pressures, it exhibits many of the characteristics that increasingly challenge LTC systems, including population ageing, dispersed settlement patterns, and growing demand for community-based care. In this study, these trends provide the macro-level basis for understanding the subsequent regional analysis of care preferences, spatial accessibility, and travel-related operational costs in the Posavje region and the Municipality of Krško.

Such demographic developments increase pressures on long-term care systems, especially regarding accessibility and service provision. Similar challenges have been observed across EU countries, particularly in rural and geographically dispersed regions, where ensuring equitable spatial access to long-term care remains a major policy concern [1,2]. In such settings, accessibility is shaped not only by service availability but also by travel distance, transport options, and the spatial distribution of providers [1,2]. Older adults in rural areas often face significant barriers related to distance, limited transportation options, and uneven service distribution [13,14,15]. These factors increase reliance on LTHC but also raise the logistical complexity and cost of service delivery. Travel time and transportation costs for care providers are not merely operational issues but are key determinants of system efficiency, accessibility, and equity [16].

Beyond organizational and economic considerations, limited accessibility and fragmented service provision can negatively affect health outcomes and quality of life. Reduced access to services and social interaction is associated with increased risks of social isolation, poorer mental and physical health, and higher use of emergency and institutional care [17,18,19,20]. This underscores the need to view long-term care not only as a service delivery issue but as a broader public health challenge [5].

In practice, these barriers result in concrete service limitations. Older adults in remote villages are left without appropriate support, leading to deterioration in their health status and overall quality of life [21]. They must move to nursing homes in cities, far from their social networks, if there is no reliable support from relatives or organized home care.

One of the main advantages of long-term home care (LTHC) is that older adults prefer to remain in their familiar environment and within their social networks, which is crucial for their well-being. However, this requires significant involvement from family members, informal care providers, and the broader community.

In Slovenia, an additional challenge is the spatial dispersion of LTC beneficiaries. This dispersion increases the complexity of organizing care and places greater demands on service providers, especially in rural areas and among older adults living alone or in households composed only of older people. For Slovenia, the numbers of those living without younger household members are provided in Table 1.

The data indicate a substantial number of older adults living in households without younger household members, particularly among the oldest age groups, which may increase reliance on formal long-term care services and mobility-dependent care provision.

The 2025 amendment to the Slovenian Long-Term Care Act (ZDOsk-1 [23]) introduced several forms of care provision, including long-term home care (LTHC), institutional services, and financial support mechanisms.

**Figure 3:**
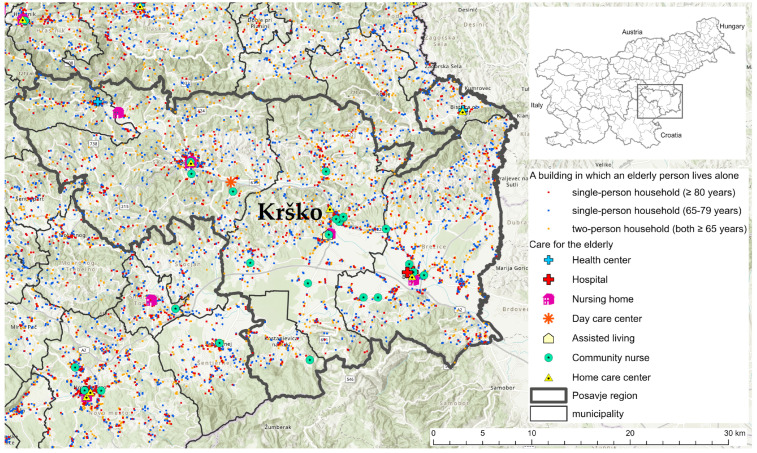
Dispersion of older adults’ households (single-person households for ages 65–79 are shown in blue, and for ages 80+ in red) in the Posavje region (which includes the Municipality of Krško) and the locations of their care centres (source: authors’ elaboration; basemap: Esri).

The study therefore examined in detail the challenges of the ageing population by spatial units in Slovenia, the spatial distribution of the population by age groups, the spatial distribution of selected facilities necessary to meet the needs of the elderly population, and the existing essential socioeconomic services for them (nursing homes, day centres for older adults, health centres, and pharmacies). It was especially important to assess the spatial accessibility of health and social care providers to older adults who need long-term care, to ensure they receive the necessary services. These analyses provide an important basis for future planning of LTC networks, including decisions regarding the spatial organization of services and the distribution of LTC providers. Particular attention was given to the Municipality of Krško and the Posavje region as a representative case of geographically dispersed LTC provision (Figure 3).

The distribution of older adults’ housing, as shown in Figure 3, together with projections of the ageing population, indicates not only a growing demand for long-term care but also the need to adapt living environments to support ageing in place. These demographic and spatial trends create additional financial pressures, particularly for individuals without adequate housing conditions or access to LTC services.

Community-based housing models, often referred to as “silver villages”, may represent one possible approach to addressing accessibility and organizational challenges in LTC provision. These models combine independent living with proximity-based care provision and shared infrastructure, potentially reducing travel distances for care providers while maintaining users’ attachment to their local environment. As such, they may offer an intermediate solution between dispersed LTHC and institutional care and have already been considered in local development initiatives in Slovenia [24].

Compared to other European countries, Slovenia has been relatively slow in developing community-based housing arrangements for older adults. Recent legislative changes, including the adoption of the Long-Term Care Act (ZDOsk) in 2021 and the revised ZDOsk-1 framework introduced in 2023, have gradually expanded the possibilities for providing LTC services in alternative and assisted housing settings [25,26,27]. These developments reflect broader discussions on adapting living environments and LTC organization to the needs of an ageing population [26].

Previous research in Slovenia has shown a strong attachment among older adults to their home environments and local communities. Specifically, 85% of respondents had never seriously considered moving from their home environments and almost two-thirds had lived at home for more than 30 years. A low percentage of respondents considered moving to alternative forms of housing, which, according to the authors, remains sufficiently high to warrant additional research [21].

Findings from previous studies, along with our fieldwork interviews, highlight the need for further research in this area. Similarly to other European countries, Slovenia faces demographic ageing, waiting lists for institutional care, workforce shortages, and unequal access to long-term care services [2]. These challenges underscore the importance of developing intermediate forms of living, such as community-based housing arrangements, intergenerational housing, and other community-based solutions, supported by appropriately located social infrastructure in both rural and urban areas.

In response to these challenges, recent policy developments in Slovenia have increasingly emphasized integrated care approaches. ZDOsk-1 envisions the development of integrated care, in line with broader EU policy directions promoting coordinated and person-centred care systems [2,5]. LTC, centred on the individual, integrates health and social services around users’ needs. It adopts a holistic perspective that considers not only medical conditions but also broader social dimensions of care [28]. User-centred care, supported by a care coordinator, is essential for ensuring continuity and quality of care, as it enables effective coordination between health services and community-based support, as reflected in local policy initiatives and pilot projects [29]. This aligns with broader conceptualizations of person-centred and holistic care that emphasize the integration of the social and organizational dimensions of care delivery [30,31]. Formal care represents a regulated and coordinated system with defined standards. However, it is expected to be increasingly complemented by informal care due to workforce shortages and demographic pressures [32], as also indicated in local LTC development initiatives [33].

Despite the growing body of literature on long-term care and integrated care models, several important gaps remain. In particular, the role of spatial factors—such as settlement dispersion, travel distances, and logistical costs—is often underexplored or only partially addressed in existing studies. Furthermore, the relationship between user preferences, spatial accessibility, and the economic sustainability of LTC systems has not been sufficiently quantified, especially in rural and regionally diverse contexts [4,32]. Existing studies also often lack detailed spatial and cost-based analyses at the regional level.

This study addresses these gaps by empirically examining the relationships between spatial accessibility, user preferences, mobility-related operational characteristics, and cost structures in long-term care provision at the regional level. It examines how to support financially sustainable and spatially coordinated LTC systems at the regional level, focusing on the Posavje region and the Municipality of Krško. By analyzing differences in care preferences, evaluating the impact of distance on service utilization, and estimating travel-related operational costs in LTHC, the study provides insights into how spatial organization and logistics affect the sustainability of LTC systems. The findings contribute to discussions on community-based and spatially coordinated long-term care planning that may also be relevant for other European regions facing similar demographic and spatial challenges.

### 1.2. Research Questions and Hypotheses

Based on the identified research gaps, this study addresses the following research questions:

**RQ1:** Are there differences in preferences for LTC across age groups?
**RQ2:** What proportion of current and potential LTC users would not accept relocation to a nursing home or community-based care facility if it is located more than 10 km from their place of residence?
**RQ3:** To what extent can savings generated by reduced travel-related operational costs contribute to covering the costs of specialized housing solutions for dependent older adults?

To address these questions, the following hypotheses were tested:

**H_1_:** Preferences for LTC services differ significantly between age groups, particularly between individuals aged 80 and above and those below 80.
**H_1a_:** Individuals aged up to 79 are more likely to prefer residence in community-based housing models (e.g., silver villages) than older individuals.
**H_2_:** Fewer than one quarter of LTHC users would accept relocation to a nursing home if it is located more than 10 km from their place of residence.
**H_3_:** Savings generated by reduced travel-related operational costs may cover a substantial share of the costs associated with specialized housing units.

## 2. Materials and Methods

### 2.1. Study Design

This study applied a mixed-methods research design combining quantitative survey data, qualitative semi-structured interviews, and spatial analysis of long-term care accessibility and mobility patterns. The quantitative component examined the living conditions, mobility, service accessibility, and care preferences of older adults and other respondents. The qualitative component explored perceived needs and challenges related to LTC provision through interviews with older adults, informal caregivers, and service providers. In addition, spatial and mobility-related analyses were used to assess the geographic dispersion and operational characteristics of home-care provision in the studied region.

The study was conducted in several complementary phases. First, quantitative survey data were collected using a structured questionnaire. Second, semi-structured interviews were carried out to gain additional insights into long-term care needs and perceived service accessibility. Third, spatial representations and mobility-related analyses were prepared to illustrate the geographic characteristics and operational burden of home-care provision.

The quantitative survey data were primarily used to address RQ1 and RQ2, related to user preferences and spatial accessibility. Qualitative interviews were used to support the contextual interpretation of long-term care needs and organizational challenges. Mobility-related operational assessment and cost estimation were used to address RQ3, concerning travel-related costs and community-based housing scenarios.

### 2.2. Quantitative Survey

The quantitative part of the study was based on a structured survey aimed at examining the living conditions, mobility characteristics, accessibility of services, and LTC needs of study participants.

#### 2.2.1. Survey Instrument

The survey was conducted using a structured questionnaire prepared in the 1 KA online survey system. The questionnaire consisted of 76 questions covering demographic characteristics, housing conditions, mobility patterns, accessibility of services, satisfaction with the living environment, social inclusion, and LTC needs and preferences.

#### 2.2.2. Data Collection

Data collection was carried out between 16 March 2022 and 23 November 2025. The questionnaire was primarily distributed online. In some cases, older respondents received assistance when completing the questionnaire due to limited digital skills or physical constraints. The survey included respondents from Slovenia, with particular emphasis on the Posavje region and the Municipality of Krško.

To reduce potential bias during assisted questionnaire completion, assistance was limited to technical and procedural support, such as reading questions aloud, clarifying the response format, or helping respondents record their selected answers. Assistants were instructed not to interpret questions on behalf of respondents and not to suggest answers.

Although assisted completion enabled the inclusion of older population groups that might otherwise have been excluded from online participation, the possibility of interviewer or assistance-related response bias cannot be entirely excluded, particularly among respondents aged 80 years or more.

The extended data collection period reflected the gradual implementation of the study activities and the use of several survey waves aimed at increasing participation among geographically dispersed and older population groups with varying levels of digital accessibility.

Because the analyzed questions concerned relatively stable preferences regarding ageing, housing, proximity, and acceptable relocation distance, no major temporal effects were expected during the data collection period. However, the authors acknowledge that legislative and service-system changes in Slovenia occurred during this period; therefore, the findings are interpreted as reflecting broad preferences and planning-relevant tendencies rather than as representing a single fixed cross-sectional moment.

#### 2.2.3. Sample

A total of 1005 individuals participated in the survey process, of which 475 questionnaires were considered sufficiently complete and valid for further analysis. For individual analyses, the number of valid responses varied according to item-level completeness; therefore, some analyses were based on a larger number of valid item responses than the number of fully completed questionnaires. The sample included respondents of different age groups and household characteristics, including older adults, informal caregivers, and other residents potentially affected by LTC accessibility and service provision.

### 2.3. Qualitative Interviews

The qualitative component of the study was conducted using semi-structured interviews to gain additional insights into LTC needs, accessibility of services, mobility-related challenges, and everyday experiences associated with ageing and care provision.

#### 2.3.1. Participants

A total of 30 semi-structured interviews were conducted. The participants were divided into three groups: older adults, informal caregivers or family members, and service providers involved in LTC. Each group included 10 participants. The inclusion of different participant groups enabled the consideration of multiple perspectives related to LTC accessibility and service provision.

#### 2.3.2. Interview Procedure

The interviews were conducted in a semi-structured format and lasted approximately 30 min on average. Interviews were conducted between 2022 and 2025, and written notes were prepared during or immediately after the interviews for qualitative interpretation purposes. The interview questions addressed topics related to housing conditions, mobility, accessibility of services, social support networks, satisfaction with the living environment, and perceived long-term care needs. The interviews were used primarily to complement the survey findings and to provide a more detailed understanding of the practical challenges associated with ageing and LTC provision in geographically dispersed areas.

#### 2.3.3. Interview Analysis

The interview material was analyzed using a qualitative thematic approach. Written interview notes were reviewed repeatedly in order to identify recurring themes, patterns, and practical concerns related to housing conditions, mobility, accessibility of services, social support, perceived safety, satisfaction with the living environment, and long-term care needs. The coding was conducted manually and followed a deductive-inductive logic: the initial analytical categories were derived from the interview guide and the research questions, while additional subthemes were identified from repeated patterns in participants’ responses.

The purpose of the qualitative analysis was not to quantify interview responses but to support the interpretation of the survey, spatial, and cost-related findings. The qualitative findings were therefore used as contextual evidence explaining how users, informal caregivers, and service providers perceive different LTC arrangements in everyday practice. No qualitative data analysis software was used; the analysis was conducted manually by the research team.

### 2.4. Mobility and Spatial Analysis

Spatial and mobility-related analyses were performed to examine the geographic dispersion of LTC provision and the operational characteristics of home-care services in the studied area.

#### 2.4.1. Mobility Patterns in Home Care

The mobility analysis focused on the actual travel patterns of home-care workers providing services in geographically dispersed areas. Daily and weekly travel routes between care recipients were considered in order to evaluate the travel-related workload, distances, time requirements, and operational costs associated with home-care provision.

Care routes were primarily organized by care workers themselves based on practical experience, local knowledge, time constraints, and everyday operational requirements.

#### 2.4.2. GIS-Based Spatial Representation

GIS tools were used primarily for the cartographic visualization and spatial representation of LTC accessibility, settlement dispersion, and mobility-related characteristics of home-care provision. Spatial data and geographic representations were used to illustrate differences in accessibility and operational burden across the studied area. Spatial representations were prepared using available geographic and settlement-related data sources.

#### 2.4.3. Verification of Selected Routes Using mTSP

In selected cases, empirically observed care routes were additionally compared with routes generated using a multi-traveling salesman problem (mTSP) approach in a GIS environment.

The purpose of this comparison was not to construct a formal optimization model for the entire care system but rather to assess the consistency of intuitively selected operational routes.

The comparison indicated that the routes selected by care workers appeared to be broadly consistent with the algorithmically suggested routes. Consequently, the evaluation of travel-related costs in this study was based on the actual operational routes used in practice.

### 2.5. Statistical Analysis

Statistical analyses were performed using descriptive and comparative statistical methods. The analysis focused primarily on frequencies, proportions, and comparisons between selected respondent groups related to LTC preferences, accessibility of services, and mobility-related characteristics. Descriptive statistics were used to summarize the demographic characteristics, living conditions, accessibility indicators, and LTC preferences of the respondents.

Chi-square (χ^2^) tests were used to examine differences in categorical variables and preferences between respondent groups.

Comparisons between selected proportions were evaluated using adjusted confidence intervals based on the Agresti–Caffo method, also referred to as the “plus-four” adjustment. This approach was used to improve interval estimation reliability, particularly in comparisons involving smaller subgroup frequencies [34].

Statistical calculations were performed using standard statistical procedures, while GIS tools were used for spatial representation, route comparison, and cartographic visualization.

## 3. Results

### 3.1. Preferences for Long-Term Care by Age

To address RQ1, differences in preferences for LTC services between respondents aged 65–79 and respondents aged 80 years or more were analyzed. Hypothesis H_1_ assumed that LTC preferences differ between these age groups. The distribution of responses is presented in Table 2.

The chi-square test indicates statistically significant differences in preferences across age groups (χ^2^ = 31.39, df = 4, *p* < 0.001), providing support for Hypothesis H_1_. Respondents aged 65–79 expressed a higher preference for community-based housing options, particularly silver villages, compared to respondents aged 80 years or more.

Table 3 presents a comparison of preferences for residence in silver villages between the two age groups.

The Agresti–Caffo method indicated a statistically significant difference in the proportion of respondents preferring residence in silver villages between the two age groups (z = 3.07, *p* < 0.01), providing support for Hypothesis H_1a_.

### 3.2. Impact of Distance on Acceptance of Institutional Care

**Figure 4:**
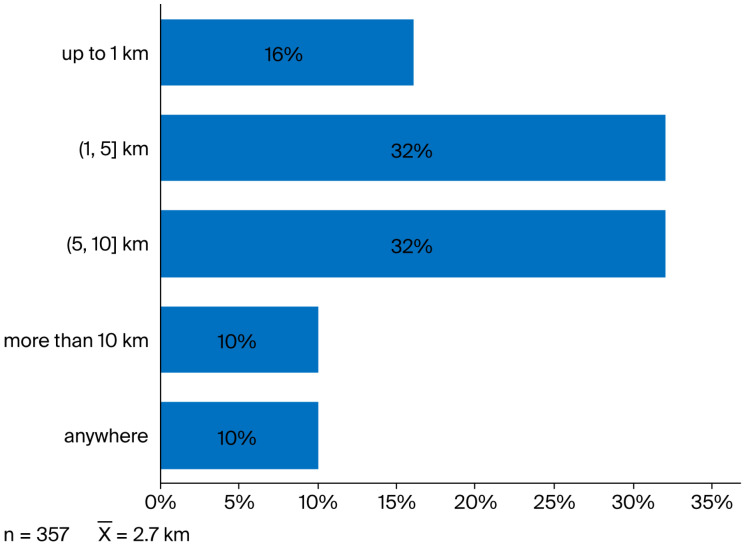
Distribution of responses to the question regarding the maximum acceptable distance of a nursing home or community-based care facility from the respondent’s residence (source: authors’ elaboration).

To address RQ2, the proportion of respondents willing to relocate to a nursing home more than 10 km from their residence was analyzed. Hypothesis H_2_ assumed that fewer than one quarter of respondents would accept relocation at such a distance. The distribution of responses is shown in Figure 4.

The results indicate that fewer than one quarter of respondents would accept relocation to a nursing home located more than 10 km from their place of residence. The Agresti–Caffo approach indicated statistical support for Hypothesis H_2_ (z = 2.38, *p* < 0.01). The findings suggest that geographic proximity remains an important factor in the acceptance of institutional and community-based long-term care arrangements.

### 3.3. Travel Costs and Cost Efficiency of LTHC

**Figure 5:**
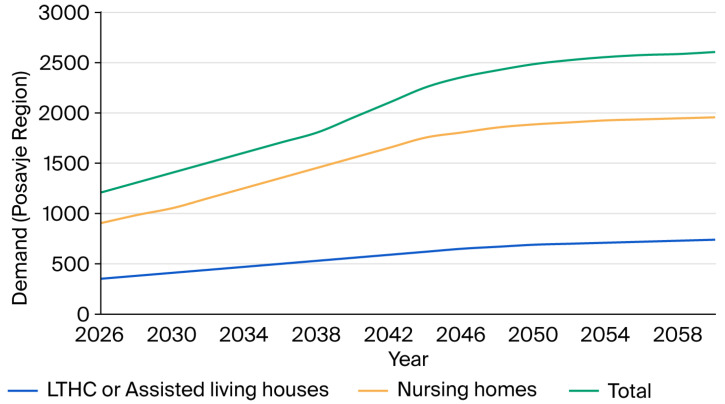
Projection of the required capacity of nursing homes and assisted living housing for the population aged 65+ for the Posavje region 2026–2060 (source: authors’ elaboration).

**Figure 6:**
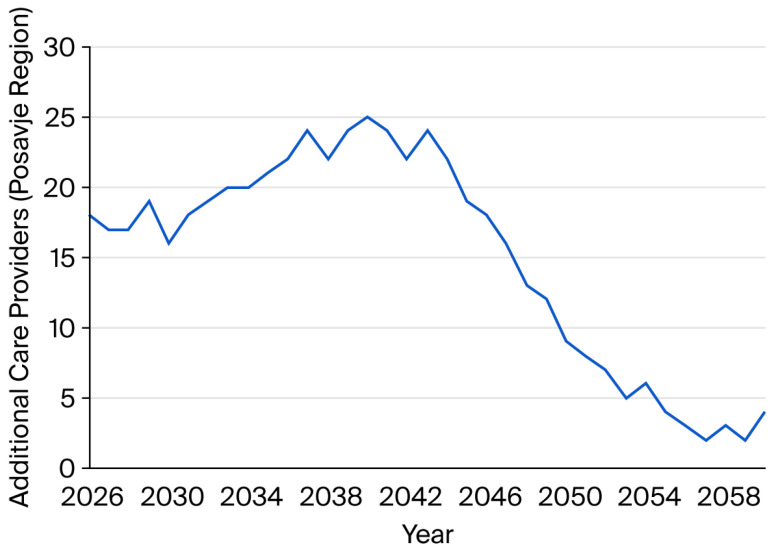
Projection of the required additional care providers per year for the population aged 65+ for the Posavje region 2026–2060 (source: authors’ elaboration).

**Figure 7:**
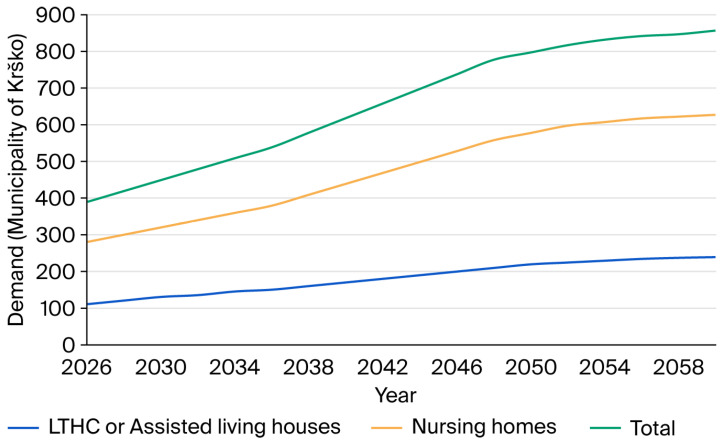
Projection of the required capacity of nursing homes and assisted living housing for the population aged 65+ for the Municipality of Krško 2026–2060 (source: authors’ elaboration).

To provide a broader contextual framework for the analysis of mobility-related costs and LTC organization, demographic projections and projected care demand in the Posavje region were additionally considered. Figure 5, Figure 6 and Figure 7 illustrate the expected future scale of LTC demand and the projected need for additional care providers in the region and in the Municipality of Krško. Although the travel-cost estimates presented in Table 4 are based on a current modelled operational scenario, the projections provide an important demographic and organizational context for interpreting the long-term sustainability implications of geographically dispersed home-care provision.

Figure 5 presents projected demand for social and healthcare services for the population aged 65 years or more in the Posavje region, while Figure 6 presents projected requirements for additional care providers.

More than 30% of the projected regional demand is expected in the Municipality of Krško. Figure 7 therefore illustrates projected LTC demand in the municipality used as the reference area for the mobility-related cost analysis presented below.

Table 4 presents the estimated travel-related costs associated with LTHC provision in the Municipality of Krško under the modelled operational scenario described above. The estimates include labour costs associated with travel time and mileage reimbursement based on average wages and standard reimbursement rates. Values are presented separately for social care and healthcare providers.

Based on Table 4, the average annual travel cost per user in the Municipality of Krško is approximately EUR 2990, which corresponds to about EUR 249 per user per month. The estimated monthly cost of housing in a specialized housing unit, such as a silver village apartment, is approximately EUR 150, including construction depreciation, energy costs, and maintenance. The comparison suggests that reductions in travel-related operational costs could partially offset the estimated costs associated with community-based housing solutions such as silver villages. Under the modelled assumptions, the estimated travel-related costs were comparable to projected housing-related costs. These findings provide indicative support for Hypothesis H_3_; however, the result should be interpreted cautiously due to the simplified assumptions of the modelled scenario and the context-specific characteristics of the studied area.

Overall, the findings suggest that spatial organization and geographic dispersion may substantially influence the operational efficiency and sustainability of LTC provision in rural and semi-rural areas.

## 4. Discussion

The findings of this study show that long-term care (LTC) planning in Slovenia cannot be addressed solely as a question of service provision but must also be understood as a spatial, logistical, and social infrastructure challenge. The results confirm significant differences in preferences for LTC services across age groups, highlighting the heterogeneity of the ageing population.

Younger older adults have a stronger preference for alternative, community-based housing models, such as silver villages and other community-based care arrangements, while the oldest age group more often prefers to remain in familiar environments or rely on conventional nursing home care. These differences indicate that LTC systems should avoid uniform solutions and instead develop diversified care models tailored to different user groups. The lack of such alternatives in the current system further explains the projected pressures on institutional capacities.

A key finding of this study is the strong attachment respondents have to their home environments and local social networks, which is closely linked to reduced risks of social isolation and better mental health outcomes [18,35]. Most respondents would not accept relocation to a nursing home or community centre if it were more than 10 km from their residence, and many preferred distances under 5 km. This confirms that spatial proximity is a critical aspect of accessibility in long-term care. In dispersed settlement systems, such as those typical of rural Slovenia, this creates a structural tension between the preference for ageing in place and the high costs of delivering long-term home care (LTHC) over long distances.

The study also shows that the sustainability of long-term care systems is closely tied to the territorial organization of services. Travel time and mileage are not minor operational factors but constitute a substantial part of total care costs. In the Municipality of Krško, the estimated average travel cost per user is approximately EUR 249 per month, based on observed operational routes used in practice and supported by comparative route assessment using the mTSP approach. When additional services such as physiotherapy and occupational therapy are included, travel costs in rural areas may exceed EUR 500 per user per month.

These findings offer valuable policy insights. Resources currently spent on dispersed travel could be partially redirected toward developing community-based housing solutions located near users’ original environments. The results suggest that the estimated costs of housing in these units may be lower than a substantial share of the travel-related operational costs associated with dispersed LTHC provision.

The promotion of small-scale, community-based housing models should not be seen as a universal substitute for LTHC. Instead, the results support a mixed model of long-term care provision that combines home care, nursing homes, and intermediate housing solutions. The appropriate model depends on individual circumstances, including health status, housing conditions, and the availability of informal support.

To complement the quantitative findings, qualitative evidence from the semi-structured interviews was used to explore perceived advantages and disadvantages of different LTC arrangements. The main themes identified from the interviews are summarized in Table 5.

As indicated by the interview findings summarized in Table 5, LTHC remains the most suitable option in many cases, particularly where users prefer to remain in their home environments. However, the high costs of delivering LTHC over long distances, especially in rural and geographically dispersed areas, represent a significant challenge. Dispersed LTHC provision increases travel time and operational costs, creating tensions between user preferences and system sustainability.

From a policy perspective, these findings raise important questions about how costs are allocated between public LTC systems and individual users. In particular, it is necessary to define the extent to which LTC insurance schemes should cover the costs of different care models, including the high travel costs associated with dispersed LTHC provision.

Although the study focused on a Slovenian regional context, similar challenges related to population ageing, dispersed settlement patterns, and LTC accessibility are also present in many rural and semi-rural areas in other European countries. The demographic trends illustrated in Figure 1 and Figure 2 suggest that the growth in the dependent older population and persistently low fertility rates are likely to increase pressure on LTC systems across Europe. Because these challenges are already observable in a country that is not among the most demographically burdened EU member states, the findings may provide useful insights for regions facing similar or even more pronounced ageing-related pressures. Consequently, the relationships identified in this study between care preferences, spatial accessibility, mobility-related costs, and the sustainability of LTC provision may also be relevant for other European regions facing comparable demographic and territorial conditions. Nevertheless, the transferability of the findings depends on differences in territorial organization, population density, transport infrastructure, and the institutional organization of LTC systems.

### Limitations

This study has several limitations. First, the survey sample may be subject to self-selection bias, as participation was voluntary and the questionnaire was primarily distributed online. Although assisted completion enabled the inclusion of older respondents who might otherwise have been excluded due to limited digital skills or physical constraints, such assistance may have influenced some responses, particularly among older participants. This risk was mitigated by limiting assistance to technical and procedural support; nevertheless, response bias cannot be fully excluded.

Second, the extended data collection period may have introduced temporal variation in respondents’ perceptions, particularly because the Slovenian LTC system was undergoing legislative and organizational changes during this period. Although the analyzed preferences relate mainly to relatively stable issues such as proximity, housing, attachment to the local environment, and acceptable relocation distance, the findings should be interpreted as planning-oriented tendencies rather than as a single time-specific measurement.

Third, the qualitative interview analysis was primarily intended to support the interpretation of the quantitative, spatial, and cost-related findings. As with most qualitative approaches, the interpretation of interview material may involve a degree of researcher subjectivity. Therefore, the qualitative findings should be understood as contextual evidence rather than as independently generalizable results.

Fourth, the findings are based on a specific regional context, with particular emphasis on the Posavje region and the Municipality of Krško. This may limit generalizability to other regions with different settlement structures, transport conditions, demographic profiles, or LTC organizational arrangements.

Finally, the cost estimates are based on current price levels and modelled operational assumptions, which may change over time. The long-term projections and the travel-cost analysis should therefore be understood as complementary analytical components: projections indicate the future scale of demand, while the cost analysis illustrates the operational implications of dispersed care provision under current modelled assumptions.

## 5. Conclusions

This study examined the relationship between user preferences, spatial accessibility, and the financial sustainability of long-term care (LTC) in Slovenia, with a particular focus on the Posavje region and the Municipality of Krško, where the spatial dispersion of LTC users is high.

The results highlight three main conclusions. First, preferences for LTC differ significantly across age groups. Younger older adults are more open to alternative, community-based housing models, while the oldest age group more frequently depends on conventional care arrangements when remaining at home is no longer feasible.

Second, spatial proximity plays a crucial role in care acceptance. A large proportion of LTC users would not accept relocation to a care facility located far from their home community, confirming the importance of maintaining connections to family, neighbourhoods, and local social networks.

Third, travel costs associated with LTHC in rural areas represent a substantial share of total LTC expenditures. The findings indicate that reductions in travel-related operational costs may offset a substantial share of the estimated accommodation costs associated with specialized housing solutions located near users’ original place of residence.

Overall, the study demonstrates that sustainable LTC systems in ageing societies require more than an expansion of service capacity and financial resources. They require spatially coordinated and organizationally sustainable care models that are both economically efficient and socially acceptable. The results suggest that community-based, spatially integrated solutions can play an important role in the future development of LTC systems in Slovenia and may also offer useful guidance for other rural and geographically dispersed regions across the European Union.

Future research should further examine operational optimization, spatial accessibility, and the long-term financial sustainability of integrated LTC systems in geographically dispersed environments.

## Figures and Tables

**Table 1:** Number of senior Slovenian citizens living alone in the house (without younger household members; 30 April 2021; source of data: [21,22]; authors’ elaboration).

Age	Single-Person Older-Adult Household		Two-Person Older-Adult Household		Three-Person Older-Adult Household	
	No. of Persons	No. of Houses	No. of Persons	No. of Houses	No. of Persons	No. of Houses
65–79 years old	27,136 (7.7%)	27,136 (4.8%)	40,324 (11.4%)	20,162 (3.6%)	1497 (0.4%)	473 (0.1%)
80 years or more	15,208 (13.0%)	15,208 (2.7%)	7224 (6.2%)	3612 (0.6%)	177 (0.2%)	51 (0.01%)
mixed 65 years or more	NA	NA	11,090 (2.4%)	5545 (1.0%)	3341 (0.7%)	1078 (0.2%)
total	42,344 (9.0%)	42,344 (7.5%)	58,638 (12.5%)	29,319 (5.2%)	5015 (1.1%)	1602 (0.3%)

Notes: NA indicates that mixed-age single-person households were not applicable within this category.

**Table 2:** Number and percentage of respondents’ preferences by age group and forms of LTC (N = 616; source: authors’ elaboration).

Age Group	At Home n (%)	Nursing Home n (%)	Home and Day Care Centre n (%)	Silver Village n (%)	Social Farm n (%)	Total n (%)
65–79 years old	130 (34.3%)	63 (16.6%)	63 (16.6%)	64 (16.9%)	59 (15.6%)	379 (100%)
80 years or more	75 (31.6%)	63 (26.6%)	63 (26.6%)	20 (8.4%)	16 (6.8%)	237 (100%)
Total	205 (33.3%)	126 (20.5%)	126 (20.5%)	84 (13.6%)	75 (12.1%)	616 (100%)

Notes: Percentages are calculated within age groups; percentages in the total row refer to the overall sample. χ^2^ = 31.39, df = 4, *p* < 0.001.

**Table 3:** Preferences for residence in silver villages by age group (n, %, N = 616; source: authors’ elaboration).

Age Group	Silver Village n (%)	Other Forms n (%)	Total n (%)
65–79 years old	64 (16.9%)	315 (83.1%)	379 (100%)
80 years or more	20 (8.4%)	217 (91.6%)	237 (100%)
Total	84 (13.6%)	532 (86.4%)	616 (100%)

Notes: Percentages are calculated within age groups; percentages in the total row refer to the overall sample. Agresti–Caffo z = 3.07, *p* < 0.01.

**Table 4:** Estimated travel-related costs for long-term home care provision in the Municipality of Krško based on a modelled operational scenario (source: authors’ elaboration).

Costs	Travel Costs Components	Social Care	Healthcare
Travel time (working hours)	Annual salary per care provider	26,400 €	24,000 €
	Salary per hour	15.53 €	14.12 €
	Weekly travel hours per care provider	7.57 h	8.8 h
	Annual travel hours per care provider	394 h	458 h
	Annual travel costs per care provider	6112 €	6460 €
Mileage costs reimbursement	Annual travel distance	16,208 km	13,670 km
	Annual mileage reimbursement costs	6970 €	5873 €
Total annual travel-related costs	Total annual travel-related costs per care provider	13,082 €	12,338 €
	Number of LTHC users per day	14	6
	Average annual travel distance per user	934 km	2056 km
	Share of working time spent on travel	23%	27%

Note: The modelled scenario included 16 care providers and 38 users. Calculations included labour costs associated with travel time and mileage reimbursement.

**Table 5:** Perceived advantages and disadvantages of different LTC arrangements derived from semi-structured interviews (source: authors’ elaboration).

Factor of Choice	Home	Nursing Home	Community-Based Housing
Independence	High, but fragile	Lowest	High and supported
Security	Dependent on house construction, location and social network	Highest	High
Help	Family, occasionally formal	Organized, permanent	When needed, optional
Mobility	The biggest challenge	Limited and costly to organize	Supported
Social	Dependent on the neighbourhood	Organized	By resident’s choice
Financial burden for society and user	High for LTHC providers and variable for users	Medium to high for users and LTC providers	Dependent on rent; lower for the LTC providers

## Data Availability

The raw data supporting the conclusions of this article will be made available by the authors on request.

## Abbreviations

The following abbreviations are used in this manuscript:

ARIS	slov. Javna agencija za znanstvenoraziskovalno in inovacijsko dejavnost Republike Slovenije (Slovenian Research and Innovation Agency)
EC	European Commission
EU	European Union
GIS	Geographical Information System
LTC	Long-Term Care
LTHC	Long-Term Home Care
MOK	slov. Mestna občina Krško (City Municipality of Krško)
mTSP	Multiple Traveling Salesman Problem
OECD	Organisation for Economic Co-operation and Development
OGRS	Official Gazette of the Republic of Slovenia
UN	United Nations
WHO	World Health Organization

## References

[1] OECD (2023). Health at a Glance 2023: OECD Indicators.

[2] EC (2024). European Commission: Directorate-General for Employment, Social Affairs and Inclusion, Affordable High-Quality Long-term Care—Catalysing Dialogue and Action Under the European Care Strategy 12 November 2024—European Commission Conference—Final Report. Publications Office of the European Union. https://data.europa.eu/doi/10.2767/0310654.

[3] Flemming J., Armijo-Olivo S., Dennett L., Lapointe P., Robertson D., Wang J., Ohinmaa A. (2021). Enhanced home care interventions for community residing adults compared with usual care on health and cost-effectiveness outcomes. Am. J. Phys. Med. Rehabil..

[4] Lobanov-Rostovsky S., Chen Y., Liu Y., Wu Y., Liu Y., Venkatraman T., French E., Curry N., Hemmings N., Bandosz P. (2023). Growing old in China in socioeconomic and epidemiological context: Systematic review of social care policy for older people. BMC Public Health.

[5] WHO, World Health Organization (2025). Integrated Care for Older People (ICOPE): Guidance for Person-Centred Assessment and Pathways in Primary Care.

[6] González-Ortiz L., Calciolari S., Goodwin N., Stein V. (2018). The core dimensions of integrated care: A literature review to support the development of a comprehensive framework for implementing integrated care. Int. J. Integr. Care.

[7] van der Feltz-Cornelis C., Attree E., Heightman M., Gabbay M., Allsopp G. (2023). Integrated care pathways: A new approach for integrated care systems. Br. J. Gen. Pract..

[8] Johri M., Béland F., Bergman H. (2003). International experiments in integrated care for the elderly: A synthesis of the evidence. Int. J. Geriatr. Psychiatry.

[9] He A., Tang V. (2021). Integration of health services for the elderly in Asia: A scoping review. Health Policy.

[10] UN, United Nations (2024). World Population Prospects 2024.

[11] Eurostat (2024). EUROPOP2024: Population Projections by Age and Sex.

[12] Eurostat (2026). Fertility Statistics. https://ec.europa.eu/eurostat/statistics-explained/index.php?title=Fertility_statistics.

[13] Genet N., Boerma W., Kringos D., Bouman A., Francke A., Fagerström C., Melchiorre M., Greco C., Devillé W. (2011). Home care in Europe: A systematic literature review. BMC Health Serv. Res..

[14] Briggs A., Valentijn P., Thiyagarajan J., De Carvalho A. (2018). Elements of integrated care approaches for older people: A review of reviews. BMJ Open.

[15] Graham H., de Bell S., Flemming K., Sowden A., White P., Wright K. (2018). The experiences of everyday travel for older people in rural areas: A systematic review of UK qualitative studies. J. Transp. Health.

[16] Szander N., McDonnell L.R., Bogataj M. (2017). Spatial dispersion of housing units as an important factor influencing long-term care operational costs. Urbani Izziv.

[17] Holt-Lunstad J., Smith T.B., Baker M., Harris T., Stephenson D. (2015). Loneliness and social isolation as risk factors for mortality: A meta-analytic review. Perspect. Psychol. Sci..

[18] Courtin E., Knapp M. (2017). Social isolation, loneliness and health in old age: A scoping review. Health Soc. Care Community.

[19] Cacioppo J.T., Cacioppo S. (2018). Loneliness in the modern age: An evolutionary theory of loneliness (ETL). Adv. Exp. Soc. Psychol..

[20] National Academies of Sciences, Engineering and Medicine (2020). Social Isolation and Loneliness in Older Adults: Opportunities for the Health Care System.

[21] Bogataj D., Drobne S., Bogataj M., Rogelj V. (2023). Geo-Gerontološki Observatorij—Posavska Regija.

[22] MNZ, Ministry of the Interior of the Republic of Slovenia (2021). Data on the Number of Residents by Age Structure in a Building with House Number as of 30 April 2021.

[23] OGRS, Official Gazette of the Republic of Slovenia (2025). Zakon o Dolgotrajni Oskrbi (ZDOsk-1). https://pisrs.si/pregledNpb?idPredpisa=ZAKO8819&idPredpisaChng=ZAKO9120.

[24] MOK, Mestna Občina Krško (2022). Na Senovem Predstavili Umestitev Pametne Srebrne Vasi. https://www.krsko.si/objava/652796.

[25] OGRS, Official Gazette of the Republic of Slovenia (2021). Zakon o Dolgotrajni Oskrbi (ZDOsk). https://pisrs.si/pregledPredpisa?id=ZAKO7621.

[26] Kerbler B., Černič Mali B. (2018). Bivanje starejših ljudi in prilagajanje grajenega okolja za funkcionalno ovirane. Kakov. Starost.

[27] OGRS, Official Gazette of the Republic of Slovenia (2023). Zakon o Dolgotrajni Oskrbi (ZDOsk-1). https://pisrs.si/pregledPredpisa?id=ZAKO8819.

[28] Colombo F., Llena-Nozal A., Mercier J., Tjadens F. (2011). Help Wanted? Providing and Paying for Long-Term Care.

[29] OGRS, Official Gazette of the Republic of Slovenia (2020). Sklep o Sofinanciranju Projekta Dolgotrajne Oskrbe v Skupnosti MOST. https://www.uradni-list.si/glasilo-uradni-list-rs/vsebina/2020-01-3517/sklep-o-sofinanciranju-projekta-dolgotrajne-oskrbe-v-skupnosti-most.

[30] Allen D. (2014). Re-conceptualising holism in the contemporary nursing mandate: From individual to organisational relationships. Soc. Sci. Med..

[31] McCormack B., McCance T. (2016). Person-Centred Practice in Nursing and Health Care: Theory and Practice.

[32] OECD (2020). Who Cares? Attracting and Retaining care Workers for the Elderly.

[33] MOK, Mestna Občina Krško (2021). V Krškem Nova Oskrbovana Stanovanja. https://www.krsko.si/objava/391914.

[34] Agresti A., Caffo B. (2000). Simple and effective confidence intervals for proportions and differences of proportions result from adding two successes and two failures. Am. Stat..

[35] Akinyemi O., Abdulrazaq W., Fasokun M., Ogunyemi O., Ikugbayigbe S., Nwosu U., Michael M., Hughes K., Ogundare T. (2025). The impact of loneliness on depression, mental health, and physical well-being. PLoS ONE.

